# Bruton’s tyrosine kinase is a possible therapeutic target in microscopic polyangiitis

**DOI:** 10.1186/s13075-023-03201-9

**Published:** 2023-11-06

**Authors:** Issei Nakade, Yuto Tamura, Fuyu Hashimoto, Yuko Ariza, Shingo Hotta, Hirofumi Fujigaya, Suishin Arai, Mai Taniguchi, Hodaka Ogawa, Yuka Nishibata, Sakiko Masuda, Daigo Nakazawa, Utano Tomaru, Akihiro Ishizu

**Affiliations:** 1 Department of Medical Laboratory Science, Faculty of Health Sciences, Hokkaido University, Kita-12, Nishi-5, Kita-Ku, Sapporo, 0600812 Japan; 2 Department of Discovery and Research, Ono Pharmaceutical Corp. Ltd., Osaka, Japan; 3 Department of Rheumatology, Endocrinology and Nephrology, Faculty of Medicine and Graduate School of Medicine, Hokkaido University, Sapporo, Japan; 4 Department of Surgical Pathology, Hokkaido University Hospital, Sapporo, Japan

**Keywords:** MPO-ANCA, MPA, NETs, Btk, Btk inhibitor, Tirabrutinib

## Abstract

**Background:**

Bruton’s tyrosine kinase (Btk) is an enzyme expressed in leukocytes other than T lymphocytes and plasma cells and involved in B-cell receptor- and Fcγ receptor (FcγR)-mediated signal transduction. Btk inhibitors potentially suppress autoantibody production due to the expected inhibitory ability of B lymphocyte differentiation into antibody-producing plasma cells and reduce FcγR-mediated neutrophil activation, including the release of neutrophil extracellular traps (NETs). Microscopic polyangiitis (MPA) is a systemic small-vessel vasculitis characterized by the pathogenic autoantibody, antineutrophil cytoplasmic antibody (ANCA) that reacts with myeloperoxidase (MPO). MPO and MPO-ANCA immune complex (IC)-induced FcγR-mediated NETs are critically involved in MPA pathogenesis. This study aimed to demonstrate the therapeutic efficacy of the Btk inhibitor tirabrutinib on MPA.

**Methods:**

Various doses of tirabrutinib or vehicle were orally administered to Sprague–Dawley rats daily. Four weeks later, the number of peripheral B lymphocytes was counted, and Btk phosphorylation in B lymphocytes was evaluated by flow cytometry. Human peripheral blood neutrophils were stimulated by MPO and anti-MPO antibody ICs (MPO and anti-MPO-ICs), and Btk and its downstream Vav phosphorylation were assessed by western blotting. The effects of tirabrutinib on MPO and anti-MPO-IC-induced NET formation were examined in vitro. Wistar Kyoto rats were immunized with human MPO to induce experimental MPA and given drug-free or tirabrutinib-containing feed (0.0037% or 0.012%) from day 0 or 28. All rats were euthanized on day 42 for serological and histological evaluation.

**Results:**

Tirabrutinib inhibited Btk phosphorylation without decreasing B lymphocytes in vivo. Neutrophil Btk and Vav were phosphorylated when stimulated with MPO and anti-MPO-ICs. Tirabrutinib suppressed MPO and anti-MPO-IC-induced NET formation in vitro and ameliorated experimental MPA in a dose-dependent manner in vivo. Although MPO-ANCA production was not affected, NET-forming neutrophils in the blood were significantly reduced by tirabrutinib.

**Conclusions:**

The Btk inhibitor tirabrutinib suppressed MPO and anti-MPO-IC-induced NET formation in vitro and ameliorated experimental MPA by reducing NET-forming neutrophils but not decreasing MPO-ANCA titer in vivo. This study suggests that Btk is a possible therapeutic target in MPA.

**Supplementary Information:**

The online version contains supplementary material available at 10.1186/s13075-023-03201-9.

## Background

Bruton’s tyrosine kinase (Btk) is an enzyme expressed in leukocytes other than T lymphocytes and plasma cells [1]. Btk is involved in signal transduction through B-cell receptor (BCR) and is essential for B lymphocyte proliferation and differentiation. Btk inhibitors potentially improve autoimmune diseases in which autoantibodies play pathogenic roles due to the expected inhibitory ability of B lymphocyte differentiation into antibody-producing plasma cells. Lupus-prone mice, which had been administered with the Btk inhibitor tirabrutinib, exhibited a reduction in serum anti-double-stranded DNA antibody titer and amelioration of immune complex (IC)-mediated glomerulonephritis [2].

Btk is involved not only in BCR signaling but also in Fcγ receptor (FcγR) signaling that regulates neutrophil functions, including phagocytosis, cytokine production, and oxidative burst [1]. Neutrophil oxidative burst is a precursor to NETosis, namely, cell death with releasing neutrophil extracellular traps (NETs) [3]. The Btk inhibitor ibrutinib has been demonstrated to suppress NET release in murine models of acute lung injury due to influenza infection [4]. During influenza infection, viruses and anti-influenza antibodies form ICs, and these ICs bind FcγR on neutrophils to induce oxidative burst leading to NETosis [5].

NETs are extracellular web-like DNA with antimicrobial substances, including myeloperoxidase (MPO), released from neutrophils that undergo NETosis [3]. Although NETs can trap and eliminate pathogenic microorganisms and play important roles in innate immunity, excessive NET formation is associated with the development of collagen vascular and autoimmune diseases, including antineutrophil cytoplasmic antibody (ANCA)-associated vasculitis (AAV) such as microscopic polyangiitis (MPA) [6].

MPA is a systemic small-vessel vasculitis characterized by the pathogenic autoantibody, MPO-ANCA. This disease develops necrotizing and crescentic glomerulonephritis (NCGN) and pulmonary hemorrhage due to alveolar septal capillaritis [7]. Upon exposure to causative environmental factors, neutrophils are primed by proinflammatory cytokines, including tumor necrosis factor (TNF)-α, or anaphylatoxin C5a that is a product of the complement alternative pathway. Primed neutrophils express MPO on the plasma membrane [8] and secrete MPO, to which MPO-ANCA binds, resulting in IC formation. After binding MPO and MPO-ANCA ICs (MPO-ANCA-ICs) to FcγRs, FcγR-mediated oxidative burst, followed by NET release, occurs. Consequently, NETs injure vascular endothelium [6]. Thus, MPA is a suitable target for Btk inhibitors, possibly suppressing autoantibody production and FcγR-mediated NET formation.

Current standard interventions in AAV, including MPA, comprise glucocorticoids (GCs) and immunosuppressive drugs, such as cyclophosphamide and rituximab [9]. Although this protocol can lead to remission in patients with AAV at a high rate, there is a risk of GC-related adverse events. Avacopan, the specific inhibitor of the C5a receptor, which can suppress neutrophil priming by C5a, exhibited promising therapeutic effects on AAV [10]. GCs might be replaced by avacopan in the near future; however, it is better to obtain more drugs that can control the disease.

Tirabrutinib is now applicable for treating relapsed and refractory central nervous system B-cell lymphoma. This study aimed to demonstrate the therapeutic potential of tirabrutinib for MPA. The results demonstrated that neutrophil Btk and its downstream Vav [11] were phosphorylated when stimulated with MPO and anti-MPO antibody-ICs (MPO and anti-MPO-ICs) and that tirabrutinib suppressed MPO and anti-MPO-IC-induced NET formation in vitro. In vivo experiments demonstrated that tirabrutinib ameliorated experimental MPA by reducing NET-forming neutrophils but not decreasing MPO-ANCA titer. This is the first preclinical study targeting Btk in MPA.

## Materials and methods

### Pharmaceutical evaluation of tirabrutinib

Tirabrutinib was orally administered to male Sprague–Dawley (SD) rats daily for 4 weeks at 3, 10, 100, and 1000 mg/kg. Methylcellulose solution (0.5%, w/v) was administered to the control group. Blood was drawn from the inferior vena cava under anesthesia. B lymphocytes (CD45RA^+^ and CD3^−^ cells) were identified by a FACSCalibur flow cytometer (BD Biosciences, Franklin Lakes, NJ, USA) and CellQuest version 3.3 (BD Biosciences). For this purpose, fluorescein isothiocyanate (FITC)-conjugated anti-CD45RA antibody (clone OX-33; BD Biosciences) and allophycocyanin-conjugated anti-CD3 antibody (clone 1F4; BD Biosciences) were used.

In other experiments, tirabrutinib was orally administered to SD rats daily for 4 weeks at 0.3, 1, 3, 10, and 30 mg/kg. Methylcellulose solution (0.5%, w/v) was administered to the control group. Rats underwent laparotomy under anesthesia and were sacrificed by exsanguination 24 h after the final dosing. Lymph node cells and peripheral blood mononuclear cells (PBMCs) were treated with phosphatase inhibitor cocktail 2 (Sigma-Aldrich, St. Louis, MO, USA), stimulated by 3 mM hydrogen peroxide (H_2_O_2_) for 10 min at 37 °C, and stained using anti-phosphorylated Btk (Btk-pY223) antibodies (Cell Signaling, Danvers, MA, USA). The mean fluorescence intensity (MFI) of Btk-pY223 in B lymphocytes (identified by gating CD45RA^+^ and CD3^−^ cells) was analyzed by flow cytometry (FCM).

### Immobilization of MPO and anti-MPO-ICs on slides with chambers

Human native MPO (4 µg/well; Elastin Products, Owensville, MO, USA) was incubated in eight-well chambers on a slide overnight at 4 °C to immobilize MPO on the slide. Alternatively, human native MPO (40 µg/well) was incubated in two-well chambers on a slide similar to immobilized MPO. After washing with phosphate-buffered saline (PBS) plus 0.05% Tween 20 (PBS-T), the slides with chambers were allowed to react with rabbit anti-human MPO polyclonal antibody (0.5 µg/well for eight-well chambers and 5 µg/well for two-well chambers; Abcam, Cambridge, UK) for 1 h at room temperature (RT). PBS-T-washed slides with chambers were used as MPO and anti-MPO-IC-immobilized slides. As a control, rabbit anti-human MPO polyclonal antibody (5 µg) was immobilized on a slide with two-well chambers.

### Neutrophil lysates

Neutrophils isolated from the peripheral blood of healthy volunteers using Polymorphprep (Serumwerk Bernburg AG, Bernburg, Germany) were resuspended in RPMI 1640 supplemented with 10% fetal bovine serum (FBS), applied to two-well chambers on MPO and anti-MPO-IC-immobilized and control slides (1 × 10^6^/well), and allowed to settle at 37 °C. Three minutes later, neutrophils were harvested and lysed in 10 µl lysis buffer for 5 min on ice. The samples were centrifuged at 10,000 rpm for 15 min at 4 °C, and the supernatants were subjected to western blotting. The time course of the neutrophil harvest was determined by preliminary assessments (data not shown).

### Western blotting

Neutrophil lysates heated under reducing conditions were applied to 7.5% sodium dodecyl sulfate–polyacrylamide gels and electrophoresed. After transfer onto a polyvinylidene difluoride membrane, the membrane was soaked in PBS-T solution containing 3% bovine albumin serum to avoid nonspecific antibody binding. Immunoblotting was conducted using anti-Btk antibody (1:1000 dilution; Cell Signaling), anti-phosphorylated Btk antibody (1:1000 dilution; Cell Signaling), anti-Vav antibody (1:3000 dilution; GeneTex, Irvine, CA, USA), and anti-phosphorylated Vav antibody (1:1000 dilution; GeneTex) as primary antibodies. These rabbit polyclonal antibodies were reacted at 4 °C overnight. After washing with PBS-T, the membrane was allowed to react with horseradish peroxidase (HRP)-conjugated anti-rabbit IgG antibody (1:10,000 dilution; Jackson ImmunoResearch, West Grove, PA, USA) as a secondary antibody for 1 h at RT. After washing with PBS-T, antibody binding was detected using a chemiluminescent substrate and ImageQuant LAS4000 (Cytiva, Tokyo, Japan).

### Suppression of MPO and anti-MPO-IC-induced NET formation by tirabrutinib

Neutrophils isolated from the peripheral blood of healthy volunteers using Polymorphprep were resuspended in RPMI 1640 supplemented with 10% FBS and reacted with tirabrutinib at concentrations of 0, 2.5, 5.0, 10, 25, 50, and 100 nM for 1 h at 37 °C. Neutrophils were applied to eight-well chambers on MPO and anti-MPO-IC-immobilized and IC-nonimmobilized slides (1 × 10^5^/well) and allowed to settle at 37 °C. Four hours later, the samples were fixed with 4% paraformaldehyde (PFA) for 15 min at RT. After removing the chambers from the slides, the samples were mounted with a 4′,6-diamidino-2-phenylindole (DAPI)-containing mounting solution (Vector Laboratories, Newark, CA, USA).

In other experiments, neutrophils pretreated with tirabrutinib were primed with 5 ng/ml TNF-α for 30 min at 37 °C. Cells were applied to eight-well chambers on MPO and anti-MPO-IC-immobilized and IC-nonimmobilized slides (1 × 10^5^/well) and allowed to settle at 37 °C. Four hours later, cells were collected and subjected to FCM to evaluate cell swelling that precedes NET formation [12]. The time course protocol was determined according to the previous study [13].

### Image analysis

Five microphotographs were taken randomly. DAPI-positive area/neutrophil was calculated using ImageJ 1.50i (https://imagej.nih.gov/ij/; National Institutes of Health, Bethesda, MD, USA).

### MPO-AAV rats

Wistar Kyoto rats (4 weeks old, male) were immunized with human native MPO to induce experimental MPA according to Little’s protocol [14]. In brief, human native MPO (1600 µg/kg) was injected subcutaneously with Freund’s complete adjuvant on day 0, and 800 ng pertussis toxin was injected intraperitoneally on days 0 and 2. Rats were given drug-free feed (*n* = 6) or tirabrutinib-containing feed (0.0037% or 0.012%) from day 0 (preadministration; *n* = 8 per group) or from day 28 (postadministration; *n* = 8/group). Body weight and food intake were measured weekly, and blood was sampled by tail cut every 2 weeks. Blood samples were heparinized, and the separated plasma was stored at − 20 °C until analysis. Urine was collected on day 40. Qualitative evaluation of hematuria and proteinuria was performed using a dipstick (Siemens Healthineers, Erlangen, Germany). All rats were euthanized on day 42 for histological and serological evaluation.

### Pathological findings

Formalin-fixed systemic organs were embedded in paraffin. Sections (3.75 µm thick) of formalin-fixed, paraffin-embedded tissues were subjected to hematoxylin-and-eosin staining. Glomeruli exhibiting NCGN, endocapillary proliferation, and tuft necrosis in the maximal section of the kidney were counted, and the glomerular lesion rate (affected glomeruli/total glomeruli) was calculated. Tubular erythrocyte casts in the renal cortex, which represented glomerular bleeding, were also counted. Microscopic foci of pulmonary hemorrhage with regional alveolar bleeding in the maximal section of the lung were counted under a low-power field of view.

### MPO-ANCA titer

MPO-ANCA titer was determined using the human MPO-immobilized enzyme-linked immunosorbent assay (ELISA) plate (Euroimmun, Lübeck, Germany) and HRP-conjugated goat anti-rat IgG antibody (Bethyl Laboratories, Montgomery, TX, USA).

### NET-forming neutrophils in the blood

Five rats in the control (drug-free) group, six rats in each preadministration group (low and high doses), three rats in the low-dose postadministration group, and four rats in the high-dose postadministration group were randomly selected and subjected to this assay. Rat polymorphonuclear cells were separated from the whole blood on day 42 using Polymorphprep. Cells were resuspended in RPMI 1640 supplemented with 10% FBS and reacted with Sytox Green, a plasma membrane-impermeable DNA-binding dye (Thermo Fisher Scientific, Waltham, MA, USA), according to the manufacturer’s instructions. Sytox Green-positive NET-forming neutrophils per 5 × 10^4^ cells in the neutrophil gate were calculated by FCM, as described previously [15].

### Immunofluorescent staining for NETs

Frozen kidney sections were subjected to immunofluorescent staining for NETs. The sections were masked with protein block serum-free solution (Agilent, Santa Clara, CA, USA) and allowed to react with rabbit anti-citrullinated histone H3 (Cit-H3) antibody (10 μg/ml; Abcam) for 60 min at RT. The sections were made to react with Alexa Flour 594-labeled donkey anti-rabbit IgG antibody (4 μg/ml; Abcam) and FITC-conjugated mouse anti-MPO monoclonal antibody (2 μg/ml; LSBio, Seattle, WA, USA) for 60 min at RT in the dark. Autofluorescence was suppressed by Vector TrueVIEW (Vector Laboratories). After mounting with a DAPI-containing mounting solution, the sections were observed under fluorescence microscopy.

### Statistical analysis

Dunnett’s and Steel’s tests were applied to parametric and nonparametric comparisons among tirabrutinib-administered and control rats. One-way analysis of variance was applied to parametric comparison among multiple groups in vitro. The Mann–Whitney *U* test was applied to a nonparametric comparison between the two groups in vitro. *p* < 0.05 was considered statistically significant.

## Results

### Tirabrutinib suppressed Btk phosphorylation without decreasing B lymphocytes

**Fig. 1:**
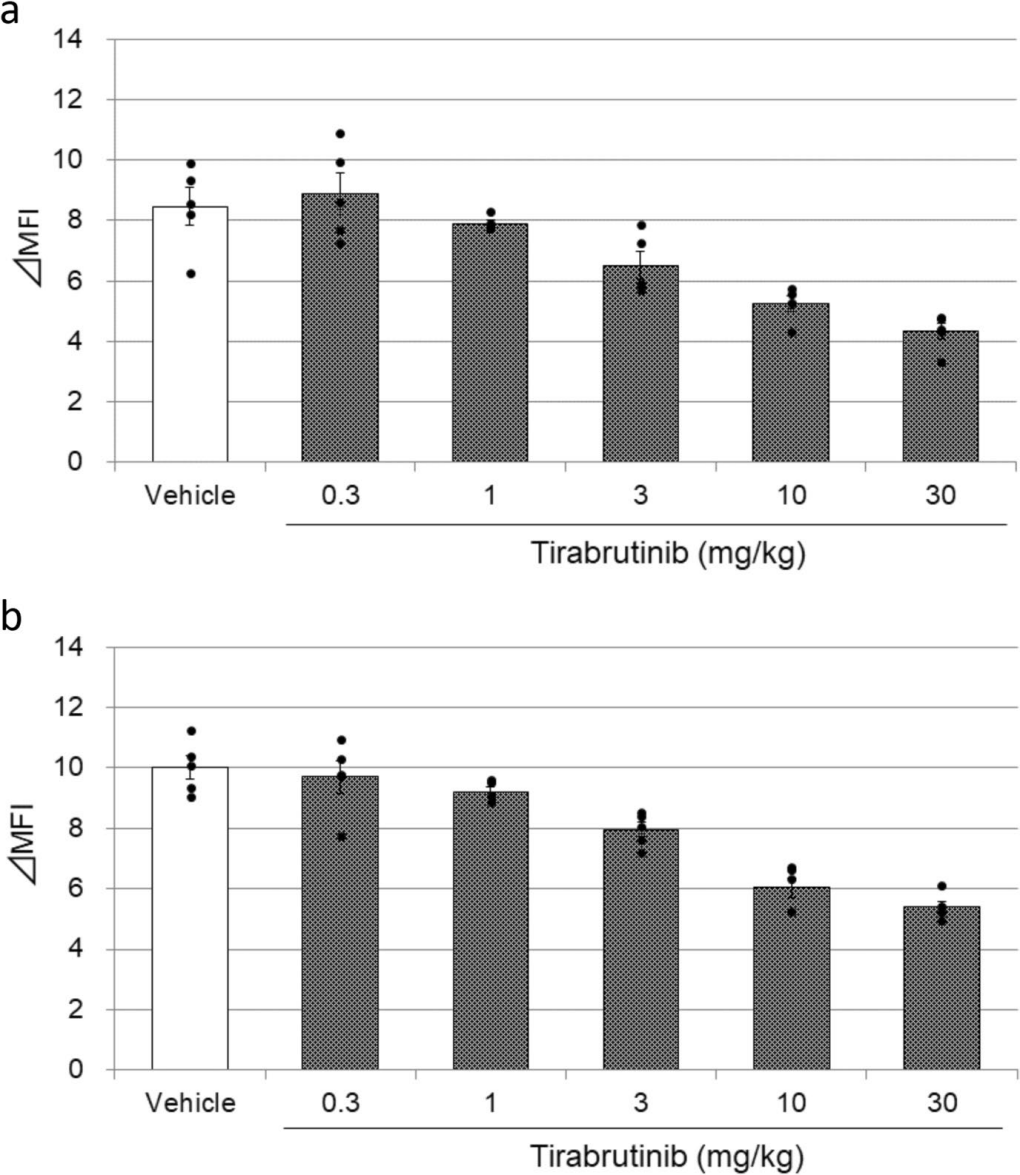
Btk phosphorylation in B lymphocytes derived from lymph node cells and PBMCs in tirabrutinib-administered rats. Various doses of tirabrutinib or vehicle were orally administered to SD rats daily for 4 weeks. Rats underwent laparotomy under anesthesia and were sacrificed by exsanguination 24 h after the final dosing. Lymph node cells (**a**) and PBMCs (**b**) were treated with phosphatase inhibitor cocktail 2, stimulated by 3 mM H_2_O_2_ for 10 min at 37 °C, and stained using Btk-pY223 antibodies. The MFI of Btk-pY223 in B lymphocytes (identified by gating CD45RA^+^ and CD3^−^ cells) was analyzed by FCM

Various doses of tirabrutinib or vehicle were orally administered to normal rats for 4 weeks. The number of B lymphocytes (CD45RA^+^ and CD3^−^ cells) was not decreased by tirabrutinib (Table 1). In contrast, tirabrutinib suppressed H_2_O_2_-induced Btk phosphorylation in lymph node cells (Fig. 1a) and PBMCs (Fig. 1b) in a dose-dependent manner. Similar findings were observed in mice transplanted with human B lymphoma cells [16]. These findings indicated that oral administration of tirabrutinib could inhibit Btk phosphorylation without decreasing B lymphocytes in vivo.

**Table 1:** B lymphocyte number in tirabrutinib-administered rat blood

	Tirabrutinib				
Dose (mg/kg/day)	0	3	10	100	1000
*N*	10	10	10	10	10
CD45RA^+^ CD3^−^ (× 10^3^/mm^3^)	2.179 ± 0.693	2.747 ± 1.063	2.278 ± 0.324	2.312 ± 0.651	2.528 ± 0.797

Values are expressed as the mean ± standard deviation

### Btk and Vav phosphorylation in neutrophils stimulated with MPO and anti-MPO-ICs

**Fig. 2:**
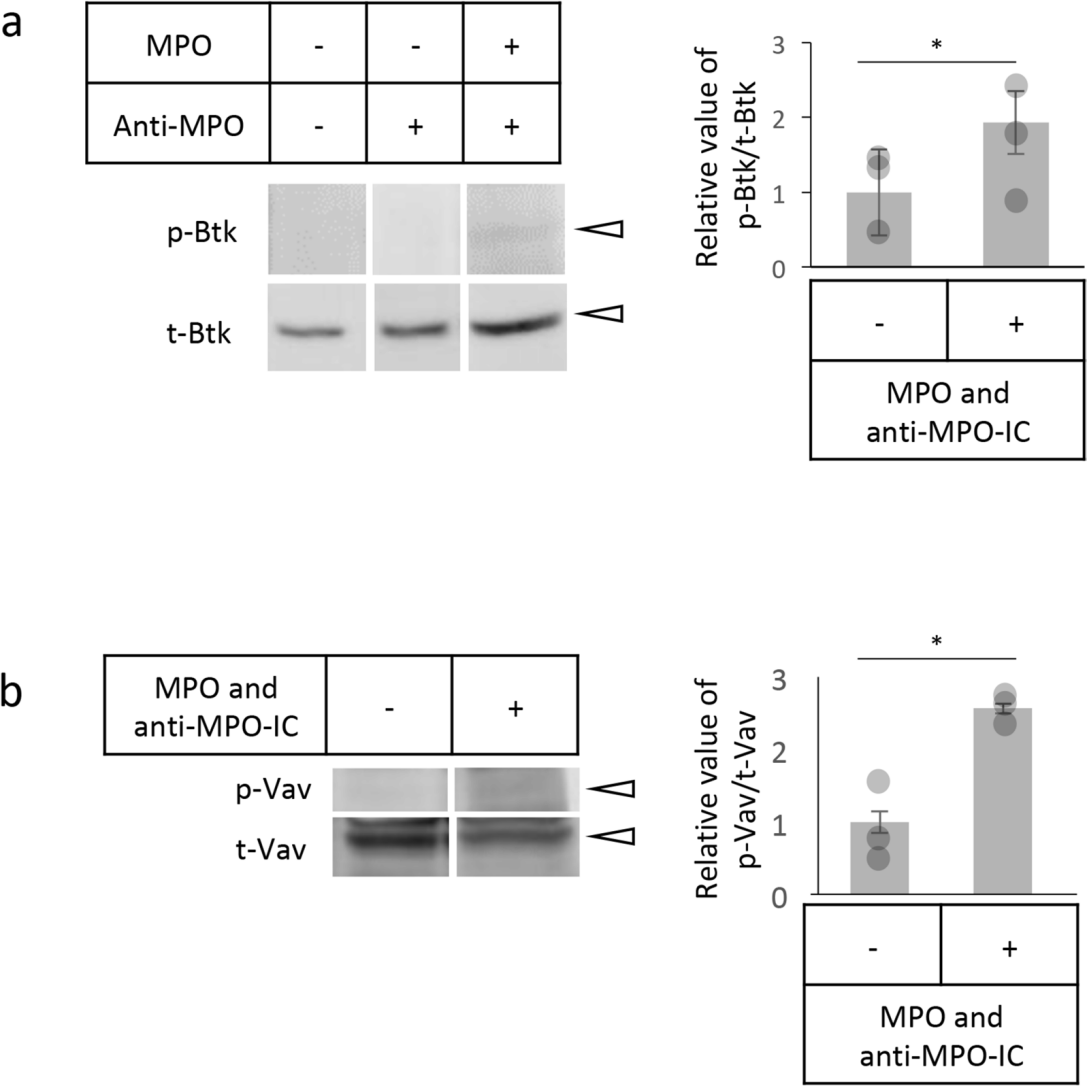
Btk and Vav phosphorylation in MPO and anti-MPO-IC-stimulated neutrophils. Human peripheral blood neutrophils were applied to two-well chambers on MPO and anti-MPO-IC-immobilized slides (1 × 10^6^/well) and allowed to settle at 37 °C. As controls, neutrophils were applied to two-well chambers on IC-nonimmobilized or anti-MPO antibody-immobilized slides (1 × 10^6^/well). Three minutes later, neutrophil lysates were collected and subjected to western blotting using anti-Btk and anti-phosphorylated Btk (p-Btk) antibodies (**a**) and anti-Vav and anti-phosphorylated Vav (p-Vav) antibodies (**b**). Because anti-Btk and anti-Vav antibodies react with both nonphosphorylated and phosphorylated antigens, each specific band was labeled as total Btk (t-Btk) and total Vav (t-Vav). Experiments were repeated thrice, the intensity of specific bands (arrowheads) was quantified using ImageJ, and the phosphorylation degree in the samples was calculated as p-Btk/t-Btk and p-Vav/t-Vav values. The value of unstimulated neutrophils was set as 1, and the relative values of MPO and anti-MPO-IC-stimulated neutrophils were shown in the graphs with error bars indicating standard deviations. *p < 0.05

Phosphorylation of Btk and its downstream Vav [11] was assessed when human peripheral blood neutrophils were stimulated with MPO and anti-MPO-ICs. Btk was phosphorylated when neutrophils were applied to the MPO-immobilized slides coated with anti-MPO antibodies but not when applied to the anti-MPO antibody-immobilized slides without MPO (Fig. 2a). This indirectly indicated that ICs were formed on the MPO-immobilized slides coated with anti-MPO antibodies. Vav was also phosphorylated when neutrophils were stimulated with MPO and anti-MPO-ICs (Fig. 2b).

### Tirabrutinib suppressed MPO and anti-MPO-IC-induced NET formation

**Fig. 3:**
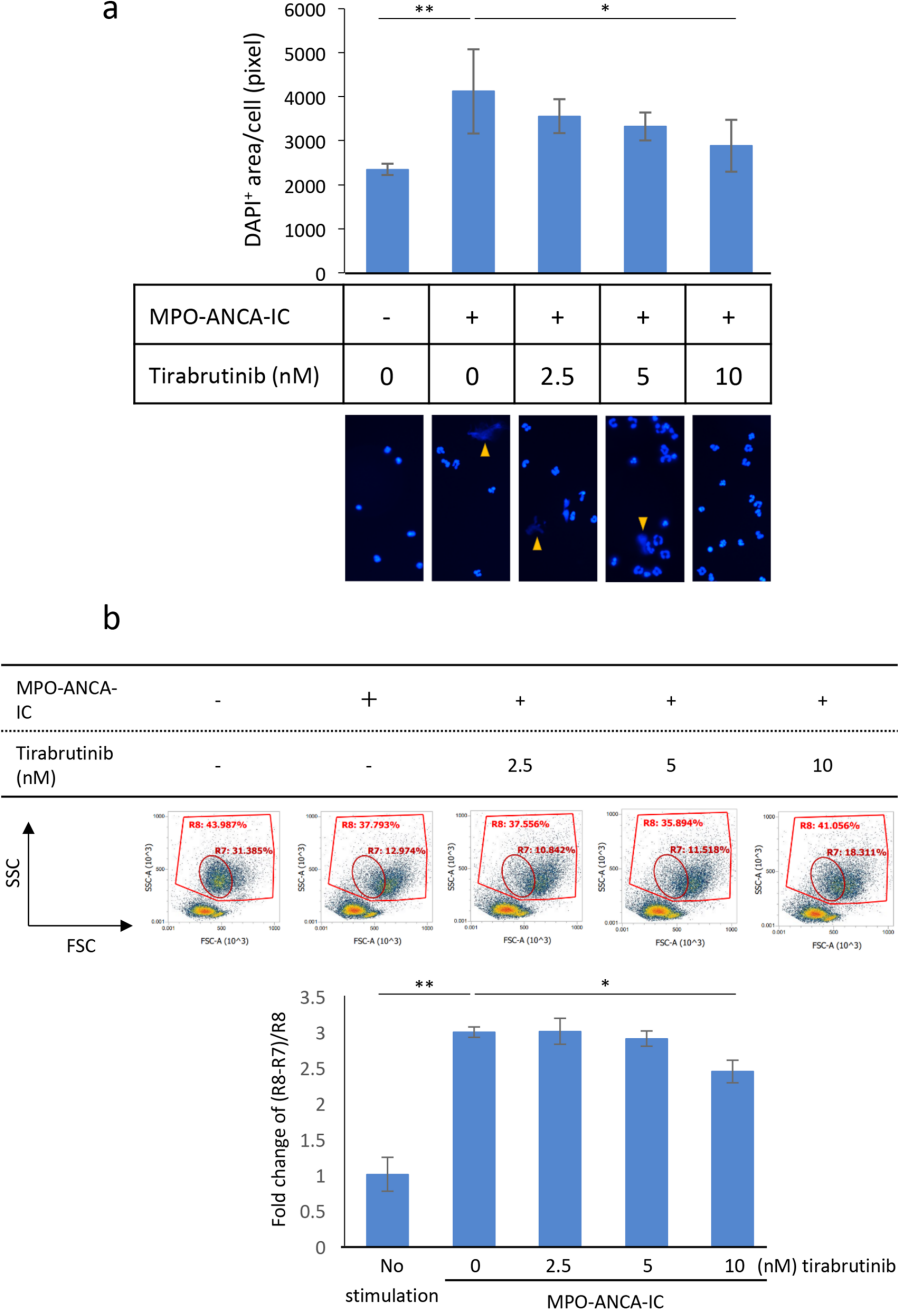
Effects of tirabrutinib on MPO and anti-MPO-IC-induced NET formation. Human peripheral blood neutrophils were allowed to react with tirabrutinib at concentrations of 0, 2.5, 5.0, and 10 nM for 1 h at 37 °C. Neutrophils were applied to eight-well chambers on MPO and anti-MPO-IC-immobilized and IC-nonimmobilized slides (1 × 10^5^/well) and allowed to settle at 37 °C. Four hours later, the samples were fixed with 4% PFA for 15 min at RT and mounted with a DAPI-containing mounting solution (**a**). Arrowheads indicate NETs. Human peripheral blood neutrophils were allowed to react with tirabrutinib at concentrations of 0, 2.5, 5.0, and 10 nM for 1 h at 37 °C, followed by 5 ng/ml TNF-α for 15 min at 37 °C. Neutrophils were applied to eight-well chambers on MPO and anti-MPO-IC-immobilized and IC-nonimmobilized slides (1 × 10^5^/well) and allowed to settle at 37 °C. Four hours later, cells were collected, resuspended in ethylenediaminetetraacetic acid-PBS, and analyzed by FCM to examine forward scatter (FSC) and side scatter (SSC) profiles (**b**). This analysis focused on swollen neutrophils [R8 gate (whole neutrophils)-R7 gate (normal size neutrophils)]. The value of samples without stimulation was set as 1. *p < 0.05; **p < 0.01

In in vitro experiments using human peripheral blood neutrophils, DAPI-positive extracellular substance was observed when neutrophils were applied to the MPO-immobilized slides coated with anti-MPO antibodies (Fig. 3a). Our previous study demonstrated that ANCA-induced DAPI-positive neutrophil extracellular substance contained Cit-H3, a marker of NETs [17]. Tirabrutinib suppressed MPO and anti-MPO-IC-induced NET formation in a dose-dependent manner, and statistically significant suppression was obtained at a concentration of 10 nM. The dose-dependent suppression of MPO and anti-MPO-IC-induced NET formation by tirabrutinib was also confirmed by FCM, focusing on neutrophil swelling that precedes NET formation [12] (Fig. 3b).

### Tirabrutinib ameliorated experimental MPA in rats

**Fig. 4:**
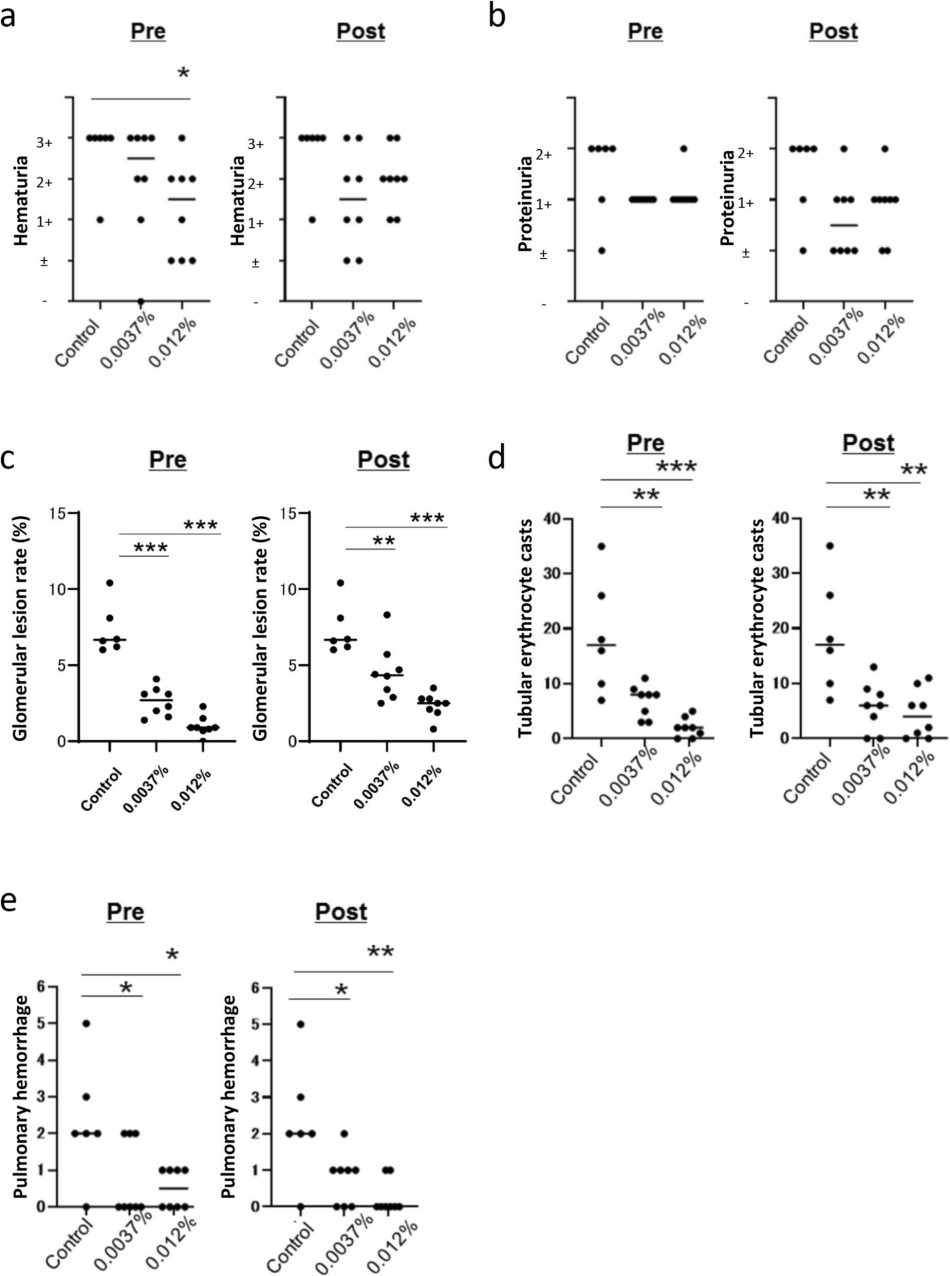
Effects of tirabrutinib on MPA model rats. MPA model rats were given drug-free feed (*n* = 6) or tirabrutinib-containing feed (0.0037% or 0.012%) from day 0 (preadministration; *n* = 8 per group) or day 28 (postadministration; *n* = 8 per group). Hematuria (**a**) and proteinuria (**b**) were determined using urine samples collected on day 40 with a dipstick. All rats were euthanized on day 42 for histological evaluation, including glomerular lesion rate (**c**), tubular erythrocyte casts in the renal cortex (**d**), and pulmonary hemorrhagic foci (**e**). *p < 0.05; **p < 0.01; ***p < 0.005

MPA model rats were given drug-free feed (*n* = 6) or tirabrutinib-containing feed (0.0037% or 0.012%) from day 0 (preadministration; *n* = 8 per group) or day 28 (postadministration; *n* = 8 per group) up to day 42. Hematuria grade was significantly decreased in the preadministration high-dose tirabrutinib (0.012%) group, with a similar trend in the preadministration low-dose tirabrutinib (0.0037%) group and the postadministration groups (Fig. 4a). Regarding proteinuria grade, there was no statistically significant difference among the groups (Fig. 4b). Pathological investigation revealed findings characteristic of MPA, including NCGN (Additional file 1: Fig. S1a), endocapillary proliferation (Additional file 1: Fig. S1b), glomerular tuft necrosis (Additional file 1: Fig. S1c), and tubular erythrocyte casts (Additional file 1: Fig. S1d) in the kidneys and alveolar hemorrhage in the lungs (Additional file 1: Fig. S1e). The glomerular lesion rate (affected glomeruli/total glomeruli; Fig. 4c), tubular erythrocyte casts in the renal cortex (Fig. 4d), and alveolar hemorrhage in the lungs (Fig. 4e) significantly decreased in a tirabrutinib dose-dependent manner in the preadministration and postadministration groups.

### Tirabrutinib did not affect MPO-ANCA production but reduced NET formation in MPA model rats

**Fig. 5:**
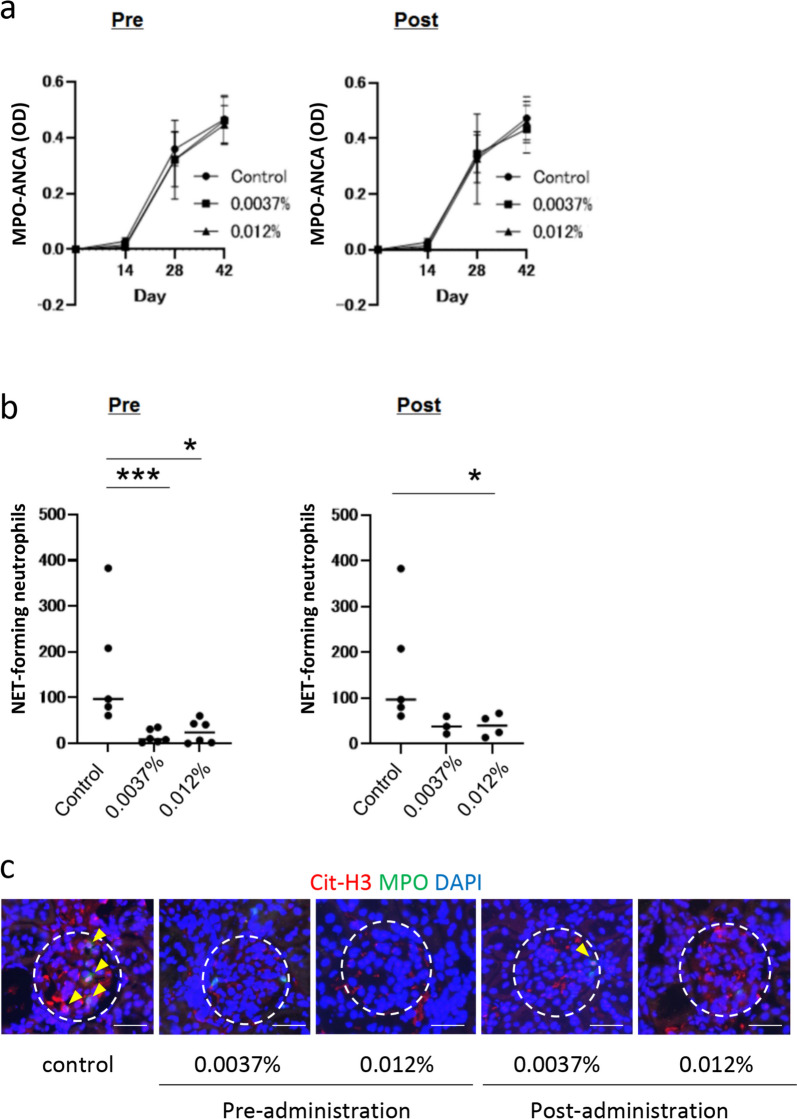
Tirabrutinib did not affect MPO-ANCA production but reduced NET formation in MPA model rats. MPO-ANCA titer in MPA model rats was quantified by ELISA chronologically (**a**). NET-forming neutrophils in the blood were counted by FCM using Sytox Green at the end of the study (**b**). Five rats in the control (drug-free) group, six rats in each preadministration group (low and high doses), three rats in the low-dose postadministration group, and four rats in the high-dose postadministration group were randomly selected and subjected to this assay. *p < 0.05; ***p < 0.005. Immunofluorescent staining for NETs in the kidneys (**c**). NETs were identified as Cit-H3, MPO, and DAPI-positive substances (arrowheads). Dotted circles indicate glomeruli. Bar, 50 µm

MPO-ANCA titer in MPA model rats was quantified by ELISA chronologically. MPO-ANCA appeared after day 14, and the titer was not affected by tirabrutinib (Fig. 5a). In contrast, NET-forming neutrophils in the blood were significantly decreased by tirabrutinib in the preadministration group even at a low dose (0.0037%) and the postadministration group at a high dose (0.012%; Fig. 5b). Immunofluorescent staining also demonstrated reduced NET deposition (Cit-H3, MPO, and DAPI-positive substances) in the kidneys of tirabrutinib-administered rats compared to controls (Fig. 5c).

## Discussion

This study demonstrated that the Btk inhibitor tirabrutinib suppressed MPO and anti-MPO-IC-induced NET formation in vitro and ameliorated experimental MPA by reducing NET-forming neutrophils but not decreasing MPO-ANCA titer in vivo. Because tirabrutinib was effective not only in the preadministration protocol but also in the postadministration protocol, it can be a therapeutic drug for MPA.

According to the involvement of Btk in BCR signaling that yields B lymphocyte differentiation into antibody-producing plasma cells [1], it was expected that MPO-ANCA production would be decreased by tirabrutinib. However, tirabrutinib did not affect MPO-ANCA production. Although the reason has been unidentified, it is consistent with a previous report demonstrating that the other Btk inhibitor evobrutinib did not reduce anti-collagen antibody production in mice with collagen-induced arthritis [18].

Tirabrutinib suppressed MPO and anti-MPO-IC-induced NET formation in a dose-dependent manner. Correspondingly, the number of NET-forming neutrophils in MPA model rats was reduced by tirabrutinib. Chen et al. reported that the pathways leading to neutrophil activation include increased intracellular calcium ions and the Srk family kinase (SFK) pathway connecting Btk and Vav [11]. In addition, Hamada et al. reported that the signal mediated through FcγRII, one of the FcγRs, phosphorylated the SFK molecule Fgr [19]. Because MPO-ANCA-ICs bind to the neutrophil FcγRII [8], MPO-ANCA-IC-mediated stimulation may activate neutrophils and release NETs via the SFK-Btk-Vav pathway. Indeed, the SFK inhibitor bletinib has been shown to suppress NET formation [20].

One of the critical problems of the current interventions using GCs and immunosuppressive drugs for AAV, including MPA, is secondary immunodeficiency-based opportunistic infection [21]. In this viewpoint, Btk inhibitors have an advantage because they do not act on T lymphocytes and plasma cells. In addition, B lymphocyte numbers and humoral immunity were not affected by tirabrutinib, as shown in this study. Btk inhibitors can be applied to patients to avoid excessive immunosuppression.

In the present in vitro study, neutrophils were stimulated by MPO and anti-MPO-ICs. There are three patterns of how ANCA binds to primed neutrophils (Additional file 2: Fig. S2a). Besides, Lelliott et al. have demonstrated that cellular adhesion is required for ANCA-induced NET formation (Additional file 2: Fig. S2b) [22]. To mimic these situations, we used MPO-immobilized slides made to form MPO and anti-MPO-ICs on the slide and then made to react with neutrophils (Additional file 2: Fig. S2c). Most antibodies are considered to bind to antigens on the slide and stimulate neutrophils via Fc and FcγR binding; however, some antibodies bridge antigens on the slide and neutrophil cell surface, and the Fc portion binds FcγR. Alternatively, the Fc portion of some antibodies may bind to the slide directly, and these antibodies crosslink antigens on the plasma membrane of neutrophils. Therefore, we believe that the major difference between the in vitro system we adopted and an assay using soluble anti-MPO antibodies is cell adhesion. As aforementioned, cell adhesion is necessary for ANCA-induced NET induction [22]; thus, we did not adopt the assay using soluble anti-MPO antibodies.

Kettritz et al. demonstrated that crosslinking of ANCA antigens induced the production of reactive oxygen species in primed neutrophils [23]. In contrast, Mulder et al. demonstrated that FcγRII was critically implicated in ANCA-induced neutrophil activation [8]. Although there remains a controversy about the pathways, Prendecki et al. recently demonstrated that a signal transducer Syk, which acts downstream of FcγRs, was activated in patients with AAV [24]. These findings suggested that ANCA-induced FcγR-mediated signals are critical for neutrophil activation in AAV pathogenesis.

The limitation of this study is the usage of a single animal model. There are two main murine MPA models: active and passive immune models [6]. In the former, Wistar Kyoto rats are immunized with human native MPO and subsequently produce anti-human MPO antibodies that cross-react with rat MPO, inducing experimental MPA [14]. In the latter, anti-mouse MPO antibodies eluted from MPO-deficient mice, which had been immunized with mouse MPO, are adopted with lipopolysaccharides into wild-type mice [25]. To elucidate the effects of Btk inhibitors on autoantibody production and the following disease development, this study employed the active immune model. However, further studies should aim to demonstrate the efficacy of tirabrutinib using the passive immune model.

## Conclusions

The Btk inhibitor tirabrutinib suppressed NET formation and experimental MPA development in rats. This phenomenon appeared to be caused by the inhibition of MPO-ANCA-IC-induced Btk phosphorylation leading to NET formation. This study suggests that Btk is a possible therapeutic target in MPA. Based on the results of this study, clinical trials of tirabrutinib for MPA are worthy of being designed.

### Supplementary Information

**Additional file 1: Fig. S1.** Representative findings in MPA model rats. MPA model rats developed glomerulonephritis with crescent formation (a; bar, 50 μm), endocapillary proliferation (b; bar, 50 μm), and tuft necrosis (c; bar, 50 μm). Tubular erythrocyte casts representing glomerular bleeding were observed in the renal cortex (d; bar, 200 μm). Pulmonary hemorrhagic lesions were counted as regional alveolar bleeding under a low-power field of view (e; bar, 200 μm).

** Additional file 2: Fig. S2.** Concept of in vitro assay. Three patterns of how ANCA binds to primed neutrophils (a). Requirement of cellular adhesion for ANCA-induced NET formation (b). MPO-immobilized slide coated with anti-MPO antibodies mimics these situations (c).

## Data Availability

The datasets gathered during manuscript preparation are available from the corresponding author upon reasonable request.

## Abbreviations

AAV: ANCA-associated vasculitis
ANCA: Antineutrophil cytoplasmic antibody
BCR: B-cell receptor
Btk: Bruton’s tyrosine kinase
Cit-H3: Citrullinated histone H3
DAPI: 4′,6-Diamidino-2-phenylindole
ELISA: Enzyme-linked immunosorbent assay
FBS: Fetal bovine serum
FCM: Flow cytometry
FcγR: Fcγ receptor
FITC: Fluorescein isothiocyanate
GC: Glucocorticoid
H_2_O_2_: Hydrogen peroxide
HRP: Horseradish peroxidase
IC: Immune complex
MPO: Myeloperoxidase
NCGN: Necrotizing and crescentic glomerulonephritis
NET: Neutrophil extracellular trap
PBMC: Peripheral blood mononuclear cell
PBS: Phosphate-buffered saline
PBS-T: PBS plus 0.05% Tween 20
PFA: Paraformaldehyde
RT: Room temperature
SD: Sprague-Dawley
SFK: Srk family kinase
TNF: Tumor necrosis factor
