# Supplementary material for: Bruton’s tyrosine kinase is a possible therapeutic target in microscopic polyangiitis
Source: Arthritis Res Ther. 2023 Nov 6;25:215. doi: 10.1186/s13075-023-03201-9 (PMC10626711; doi:10.1186/s13075-023-03201-9)
Supplement: Supplementary file 1 — Additional file 1: Fig. S1. Representative findings in MPA model rats. MPA model rats developed glomerulonephritis with crescent formation (a; bar, 50 μm), endocapillary proliferation (b; bar, 50 μm), and tuft necrosis (c; bar, 50 μm). Tubular erythrocyte casts representing glomerular bleeding were observed in the renal cortex (d; bar, 200 μm). Pulmonary hemorrhagic lesions were counted as regional alveolar bleeding under a low-power field of view (e; bar, 200 μm). [file 13075_2023_3201_MOESM1_ESM.pdf]

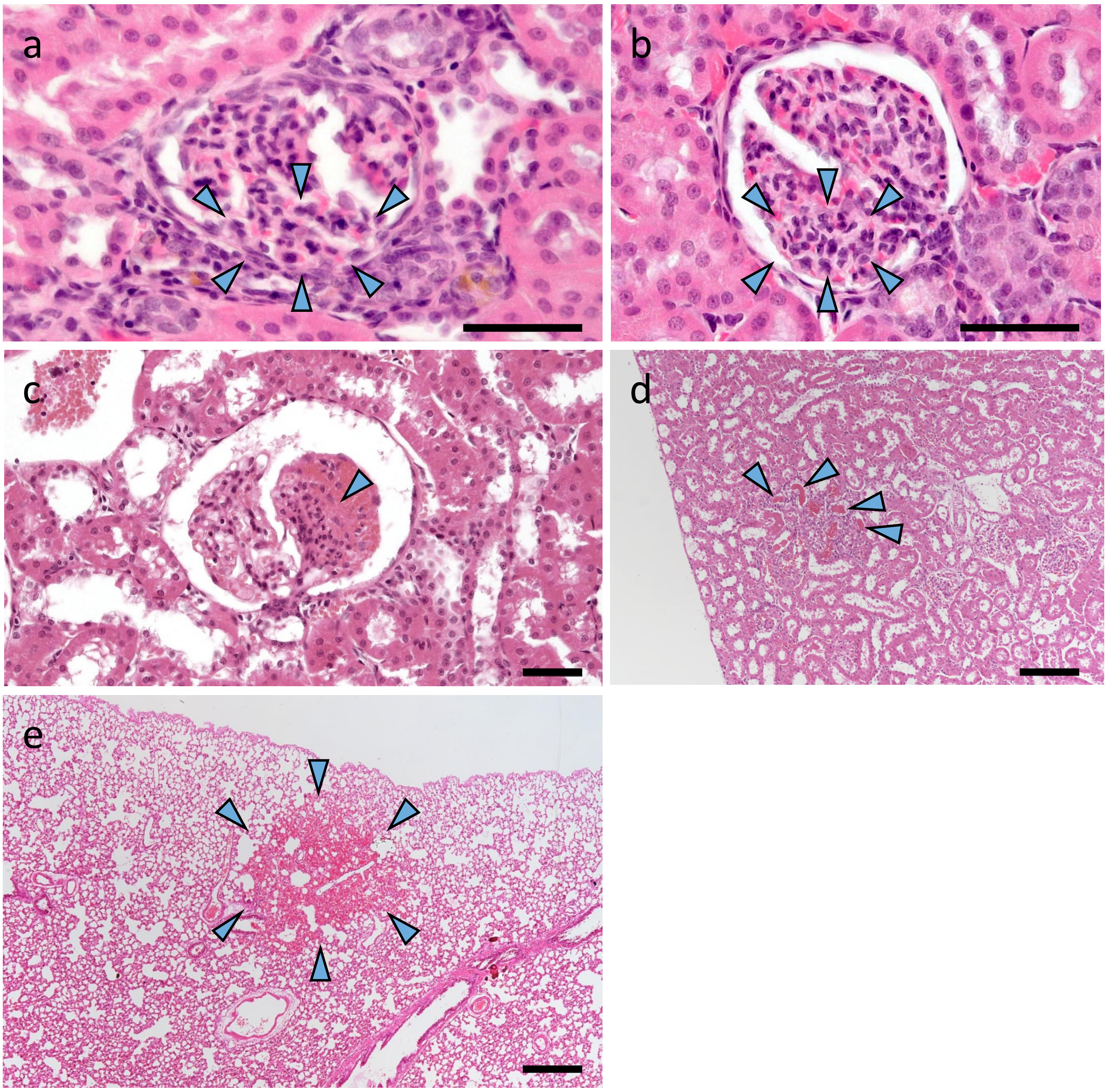

Figure S1. Representative findings in MPA model rats.

MPA model rats developed glomerulonephritis with crescent formation (**a**; bar, 50 μm), endocapillary proliferation (**b**; bar, 50 μm), and tuft necrosis (**c**; bar, 50 μm). Tubular erythrocyte casts representing glomerular bleeding were observed in the renal cortex (**d**; bar, 200 μm). Pulmonary hemorrhagic lesions were counted as regional alveolar bleeding under a low-power field of view (**e**; bar, 200 μm).
