# Supplementary material for: Bruton’s tyrosine kinase is a possible therapeutic target in microscopic polyangiitis
Source: Arthritis Res Ther. 2023 Nov 6;25:215. doi: 10.1186/s13075-023-03201-9 (PMC10626711; doi:10.1186/s13075-023-03201-9)
Supplement: Supplementary file 2 — Additional file 2: Fig. S2. Concept of in vitro assay. Three patterns of how ANCA binds to primed neutrophils (a). Requirement of cellular adhesion for ANCA-induced NET formation (b). MPO-immobilized slide coated with anti-MPO antibodies mimics these situations (c). [file 13075_2023_3201_MOESM2_ESM.pdf]

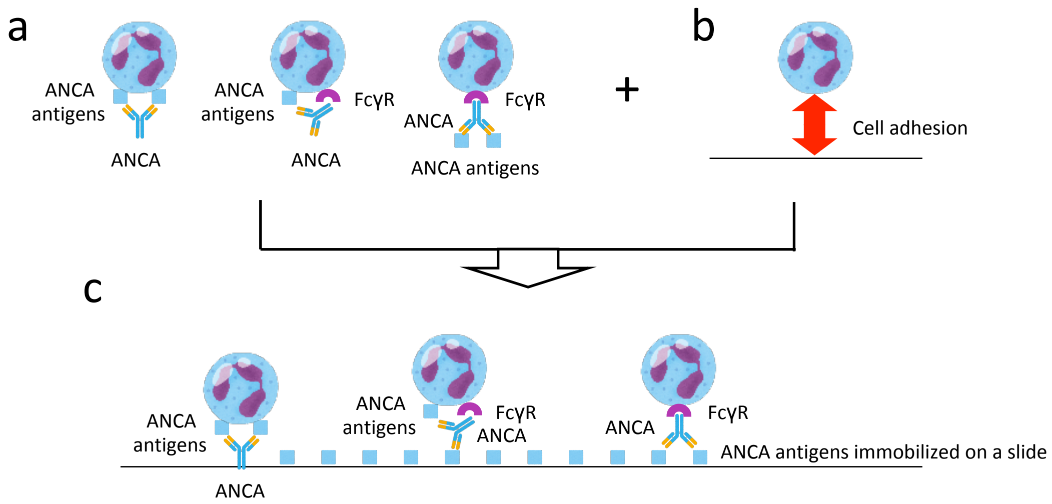

Figure S2. Concept of *in vitro* assay.

Three patterns of how ANCA binds to primed neutrophils (**a**). Requirement of cellular adhesion for ANCA-induced NET formation (**b**). MPO-immobilized slide coated with anti-MPO antibodies mimics these situations (**c**).
